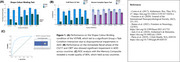# Transcultural memory markers for Alzheimer's Disease: Evidence from the ReDLat project

**DOI:** 10.1002/alz70857_106530

**Published:** 2025-12-25

**Authors:** Mario A A Parra, Martin Alejandro Bruno, Claudia Duran‐Aniotz, Andrea Slachevsky, María Isabel Behrens, Leonel Tadao Takada, Elisa de Paula, França Resende, Nilton Custodio, David Aguillon, José Alberto Ávila Funes, Agustin Ibanez

**Affiliations:** ^1^ University of Strathcylde, Glasgow, Glasgow, United Kingdom; ^2^ Instituto de Ciencias Biomédicas (ICBM) Facultad de Ciencias Médicas, Universidad Catoóica de Cuyo, San Juan, San Juan, Argentina; ^3^ CONICET, San Juan, Argentina; ^4^ ReDLat, San Juan, Argentina; ^5^ Latin American Brain Health Institute (BrainLat), Universidad Adolfo Ibañez, Santiago, Chile; ^6^ Cognitive Neurology and Dementia, Neurology Department, Hospital del Salvador, Santiago, Chile; ^7^ Neurology Department, Hospital del Salvador, University of Chile, Santiago, Región Metropolitana de Santiago, Chile; ^8^ Memory and Neuropsychiatric Clinic (CMYN), Neurology Service, Hospital del Salvador and Faculty of Medicine, Universidad de Chile, Santiago, Chile; ^9^ Geroscience Center for Brain Health and Metabolism (GERO), Santiago, Chile; ^10^ Servicio de Neurología, Departamento de Medicina, Clínica Alemana‐Universidad del Desarrollo, Santiago, Chile; ^11^ Centro de Investigación Clínica Avanza (CICA), Hospital Clínico Universidad de Chile, Santiago, Chile; ^12^ Hospital Clínico de la Universidad de Chile, Santiago de Chile, Chile; ^13^ Cognitive and Behavioral Neurology Unit, Hospital das Clinicas HCFMUSP, Faculdade de Medicina, Universidade de Sao Paulo, Sao Paulo, Sao Paulo, Brazil; ^14^ Faculdade de Medicina de Ciências Médicas de Minas Gerais, Belo Horizonte, Brazil; ^15^ Global Brain Health Institute (GBHI), University of California San Francisco (UCSF); & Trinity College Dublin, San Francisco, CA, USA; ^16^ IPN, Lima, Peru; ^17^ Grupo de Neurociencias de Antioquia, Universidad de Antioquia, Medellin, Colombia; ^18^ Instituto Nacional de Ciencias Médicas y Nutrición Salvador Zubirán, Mexico City, DF, Mexico; ^19^ Cognitive Neuroscience Center (CNC), Universidad de San Andrés, Buenos Aires, Buenos Aires, Argentina; ^20^ Global Brain Health Institute (GBHI), University of California San Francisco (UCSF); & Trinity College Dublin, Dublin, Leinster, Ireland; ^21^ National Scientific and Technical Research Council (CONICET), Buenos Aires, Argentina

## Abstract

**Background:**

Alzheimer's disease dementia (ADD) is diagnosed based on evidence of significant cognitive decline from a previous level of performance in one or more cognitive domains (DSM‐5). Jack Jr et al. (2024) acknowledged that clinical judgment remains crucial when biomarkers are discordant with clinical impressions or non‐accessible. However, available assessments lack precision in detecting ADD and are influenced by sociocultural factors, thus calling for cross‐cultural harmonization strategies (Franzen et al., 2020). The Visual Short‐Term Memory Binding Test (VSTMBT) has shown cross‐cultural validity across six European countries (Costa et al., 2017; Parra et al., 2019). We investigated whether this and other promising tests are valid across six Latin American Countries.

**Methods:**

ADD patients (*n* = 426) and Healthy Controls (*n* = 869) were recruited from Argentina, Brazil, Chile, Colombia, México and Perú. They were assessed using a comprehensive protocol (Ibanez et al., 2021), which included the VSTMBT, the Craft Story 21 Test (CS21T) and the Benson Complex Figure Test (CBFT). We used General Linear Models (GLM) to compare data across Groups and Countries controlling for confounders. We explored the classification accuracy of these tests using ROC analysis.

**Results:**

Whole‐sample VSTMBT data entered a mixed GLM revealing the traditionally reported Group (ADD vs HC) by condition interaction (*F*(1,1287) = 4.62, *p* = 0.032), driven by disproportional Shape‐Color Binding deficits. When this test, the CS21T and CBFT immediate recall data entered GLMs with Group and Country as fixed factors and age, sex, education, number of languages, and comorbidities as covariates, the main effects and the interaction proved significant. The three tests revealed significant deficits in ADD relative to HC (all *p* < 0.05). ROC analysis showed a reliable classification accuracy for a memory composite that combined the three tests (AUC: Argentina=1.0, Brazil=0.99, Chile=0.96, Colombia=0.91), México=0.91, and Perú=0.78).

**Conclusions:**

The VSTMBT, CS21T and CBFT proved reliable for assessing ADD across six LAC. Although variability was observed across countries, they revealed significant deficits in ADD, which were unaccounted for by relevant confounding factors. The composite score achieved excellent classification accuracy for most countries. The cross‐cultural validity of the VSTMBT previously reported across EU countries holds for LAC.